# Association Analysis of Somatic Copy Number Alteration Burden With Breast Cancer Survival

**DOI:** 10.3389/fgene.2018.00421

**Published:** 2018-10-01

**Authors:** Linfan Zhang, Nikta Feizi, Chen Chi, Pingzhao Hu

**Affiliations:** ^1^Department of Biochemistry and Medical Genetics, University of Manitoba, Winnipeg, MB, Canada; ^2^Centre for Healthcare Innovation, Winnipeg Regional Health Authority and University of Manitoba, Winnipeg, MB, Canada; ^3^Department of Electrical and Computer Engineering, University of Manitoba, Winnipeg, MB, Canada; ^4^Department of Computer Science, University of Manitoba, Winnipeg, MB, Canada

**Keywords:** copy number alteration, genetic burden, prognosis biomarker, breast cancer, stratified model

## Abstract

The increasing prevalence of diagnosed breast cancer cases emphasizes the urgent demand for developing new prognostic breast cancer biomarkers. Copy number alteration (CNA) burden measured as the percentage of the genome affected by CNAs has emerged as a potential candidate to this aim. Using somatic CNA data obtained from METABRIC (Molecular Taxonomy of Breast Cancer International Consortium), we implemented Kaplan-Meier estimators and Cox proportional hazards models to examine the association of CNA burden with patient’s overall survival (OS) and disease specific survival (DSS). We also evaluated the association by considering patients’ age and tumor subtypes using stratified Cox models. We delineated the distribution of CNA burden in sample genomes and highlighted chromosomes 1, 8, and 16 as the carriers of the highest CNA burden. We identified a strong association between CNA burden and age as well as CNA burden and breast cancer PAM50 subtypes. We found that controlling the effects of both age (bound by 45-year) and PAM50 subtypes on patient survival using stratified Cox models, would still result in significant association between CNA burden and patients overall survival in both Discovery and Validation data. The same trend was observed in disease specific survival when only PAM50 subtypes were controlled in the stratified Cox models. Our analysis showed that there is a significant association between CNA burden and breast cancer survival. This result is also validated by using TCGA (The Cancer Genome Atlas) data. CNA burden of breast cancer patients has a considerable potential to be used as a novel prognostic biomarker.

## Introduction

With a global estimate of 1.7 million newly diagnosed cases, breast cancer continues to be the most common malignancy affecting women worldwide. Of these, 80–90% of all breast cancer cases are sporadic. Sporadic breast cancers are non-hereditary and are believed to arise from gene damages due to multifactorial causes such as environment, aging and diet. With advances in next generation sequencing (NGS) and expression profiling studies, breast cancer is now understood as a collection of highly heterogeneous diseases with distinct clinical and molecular phenotypes (luminal A, luminal B, HER2-enriched and basal), each leading to unique clinical outcomes in terms of patients’ survival, disease progression rate, and treatment responses (Kalimutho et al., 2015). Although being highly influenced by mutations in oncogenes such as ErbB2 and tumor suppressors such as TP53 and BRCA1/2, breast tumors are mainly governed by amplifications, deletions or rearrangements of chromosomal segments (i.e., copy number alterations, CNAs) rather than mutations in a single gene (Ciriello et al., 2013).

Accumulated somatic mutations such as CNAs, single nucleotide substitutions, and translocations appear to have an important role in determining cancer progression (Futreal et al., 2004). Generally, somatic mutations are defined as non-heritable genetic alterations that occur in somatic cells. Errors in DNA duplications, exposure to chemical agents, and UV radiation are among the most common triggers of somatic mutations. Somatic alterations in the copy numbers of a DNA sequence, in the form of either gain or loss, are known as CNAs which are common in many cancer types (Beroukhim et al., 2010). Identification of recurrent CNAs (Baudis, 2007; Mitelman et al., 2009), which are reported to be strongly associated with clinical phenotypes has resulted in the discovery of new therapeutic options, targeting the identified causal mutations in many cancer types (Eder et al., 2005; Zender et al., 2006; Weir et al., 2007; McLendon et al., 2008; Chitale et al., 2009; Chapman et al., 2011; Nguyen and Neal, 2012). Accordingly, CNAs identified in breast cancer patients could also be regarded as potential biomarkers providing a considerable opportunity for therapeutic interventions (Stuart and Sellers, 2009).

It has been reported that 85% of the variations in gene expressions of breast tumors are due to somatic CNAs at gene loci (Curtis et al., 2012). It is noteworthy that CNAs often involve oncogenes and tumor suppressors (i.e., driver genes), which can directly affect cancer development and disease progression. For instance, ZNF703 has been known as an independent prognostic factor for luminal B breast cancer. According to findings from one study (Holland et al., 2011), patients with CNAs in this specific gene seem to have worse clinical outcomes. Similarly, CNAs in 3q26.2-q29, 3p26.3-p11.1, 17p13.3-p11.2, and 9p13.3- p13.2 have been deemed as predictors of lung cancer (van Boerdonk et al., 2011). Accordingly, incorporating CNA analysis of breast tumors with molecular profiling and survival outcomes of the disease can offer novel therapeutic insights.

Attempts for explaining the effects of CNAs on the advent of schizophrenia revealed that investigating CNAs in a single gene locus may not be as optimal as studying the total burden of genes influenced by CNAs (Rees et al., 2013). This can be due to a low number of identified causal genes, as well as the observation that a CNA in a single gene may affect individuals differently, based on their genetic and environmental backgrounds (Pocklington et al., 2015).

A percentage of the genome that is affected by CNAs is known as CNA burden. Several studies have reported the existence of an association between CNA burden and tumor attributes such as tumor grade, recurrence, and metastasis (Baca et al., 2013). A prostate cancer study showed that CNA burden can be regarded as a prognostic biomarker associated with cancer biochemical recurrence and metastasis (Hieronymus et al., 2014). Also, a recent lung cancer study revealed that squamous cell carcinoma lung cancer patients have an increased copy number burden in most of the individual chromosomes, particularly in the length of 50 kb (Yang et al., 2015). In consistence, investigations about the role of CNAs in patients with malignant melanomas presented an association between CNA and poor outcomes (Hirsch et al., 2013). All these studies imply the importance of assessing CNA burden in different cancers. To our knowledge, to date, CNA burden is not well-studied in breast cancer patients. In this study we aim to analyze the CNA burden on a per-individual base. We hypothesize that CNA burden (high/low) may act as a prognostic factor in associating with the survival outcome of breast cancer patients.

## Materials and Methods

### Materials

#### METABRIC (Molecular Taxonomy of Breast Cancer International Consortium) Data

Clinical annotations (including patients’ overall survival and cancer-specific survival, age at diagnosis, tumor subtype and grade, etc.) and somatic CNA profiles for Discovery (980) and Validation (985) sets of primary breast tumors were derived from METABRIC (Curtis et al., 2012). The study was obtained by permission from the METABRIC. We downloaded the data from European Genome-phenome Archive at http://www.ebi.ac.uk/ega/ under the accession number of EGAS00000000083. The patient-specific somatic CNA profiles include start and end position of each CNA, type of the CNA (gain or loss) and number of its probes (SNP and CNV probes).

We defined a somatic CNA segment as a DNA segment that is 1 kilobase or larger at variable copy numbers (gain or loss. See details below) when referred to a reference genome. The HapMap and normal datasets were used to estimate the frequency of germline copy number variations (CNVs) in the cohort, while the tumor samples were used for estimating somatic CNAs. After computing the log2 ratios for each probe, samples were segmented using the circular binary segmentation (CBS) algorithm implemented in the DNAcopy R Bioconductor package and individual patient level CNVs were called. For the tumor samples, any segmented mean that fell within a region included in the HapMap + Normals CNV list was labeled as an inherited CNV. In order to remove all possible germline CNVs, the frequencies of somatic CNAs in the tumor samples were obtained after removing the germline CNVs from the normalized pool reference. Neutral LOHs were also excluded in the data analysis. For calling alterations, the thresholds for gains and losses were set to +2 σ and -2.5 σ (σ is the standard deviation of the log2 ratio for each array) respectively. The asymmetry in the thresholds results from the assumption that one copy gain is 3/2 whereas one copy loss is 1/2. Please note that this was carried out in the original METABRIC study.

#### TCGA (The Cancer Genome Atlas) Data

To further validate our approach, it was also applied to analyze TCGA breast cancer data (The Cancer Genome Atlas, 2012). The study included 825 primary breast cancers. The clinical annotations included patients’ overall survival, age at diagnosis, tumor subtype and grade, etc. However, only 482 of the samples had PAM50 subtype information. Somatic CNA profiles were performed in the TCGA study. We used the same cutoff to define somatic CNA segments as applied in the METABRIC study.

### Methods

#### CNA Burden Definition

The total genomic regions spanned by continuous somatic CNA segments with a size of at least 1 kb identified by 5 or more probes were summed up, and the final CNA percentage was calculated based on the size of the autosomal human genome (chromosomes 1–22). In regression models, this value was referred to as the continuous CNA burden. The resulting somatic CNA burden was used to stratify breast cancer patients into high and low CNA burden groups based on the median CNA burden observed across the breast cancer genomes in METABRIC Discovery and Validation data and TCGA data, respectively. The stratified results were referred to as binary CNA burdens. We used one-sample Wilcoxon signed rank sum test to evaluate whether a given chromosome has significantly higher somatic CNA burden than others.

#### Gene Set Enrichment Analysis

For the chromosomes with significantly higher somatic CNA burden than other chromosomes, we extracted a list of genes from the somatic CNA regions on the chromosomes. We filtered the top 10% most frequent genes on each chromosome separately for loss and gain groups. We conducted our gene-set enrichment analyses using the most recently updated version of Enrichr tool (Kuleshov et al., 2016).

### Statistical Analyses

All the statistical analyses were performed using R version 3.3.1. For clinical characteristics, *p*-values were determined by Wilcoxon rank sum test for continuous variables and Fisher’s exact test for categorical variables. The associations of CNA burden with age and PAM50 subtypes were determined by linear regression and one-way ANOVA. For the METABRIC data, survival analysis was performed on breast cancer’s disease-specific survival (DSS), defined as the time period from a diagnosis to a breast cancer-related death, as well as overall survival (OS), defined as the time period from a diagnosis to a death from any cause. For the TCGA data, survival analysis was only performed on patients’ OS since the study did not provide DSS. Kaplan–Meier (KM) estimators and Cox proportional hazard (PH) models were used for the survival analysis to evaluate the association of CNA burden with OS and DSS respectively. The R package *survminer* (Kassambara et al., 2016) was used for generating the KM plots (based on *p*-values from log-rank tests indicating the significance of the differences between groups) as well as the risk-table (demonstrating the number of patients at risk at each time point). We first included CNA burden as a covariate in a Cox proportional hazard model, subsequently added age and PAM50 subtypes separately as confounding factors, and lastly generated models including all these variables. The assumption of proportional hazard was evaluated based on correlation between time and Schoenfeld’s partial residuals (Harrell, 2001). Time dependent variables (known to violate proportional hazard assumption) were assessed by stratified Cox models which evaluated the association of CNA burden with OS and DSS respectively. Firstly, the data was stratified by time dependent variables. Age is a continuous and time dependent variable, hence in our analysis we stratified it by 45-year bound. Afterward, Cox model was fitted for each category with different baseline hazard function and the same parameters for covariates (Therneau and Grambsch, 2000). All Cox model related analyses were calculated via R package *survival* (Therneau, 2015).

## Results

### METABRIC Patients’ Characteristics

The characteristics of patients from Discovery and Validation groups are compared in **Table 1**. The characteristics include age at diagnosis, CNA burden, PAM50 subtypes, grade and the status of ER, PR and Her2 defined based on the gene expression data. At the significance level of 0.05, there was not a significant difference in “the age at diagnosis” between the two groups (*p*-value = 0.21). However, patients in the Validation group showed to carry higher CNA burden than those in Discovery group (*p*-value = 0.01). The Validation group also included a greater proportion of patients with normal-like and basal-like subtypes, while the Discovery group included a greater proportion of patients with the subtype luminal A. While the two groups showed no significant difference in PR-expr (*p*-value = 0.93) and HER2-expr (*p*-value = 0.19), the expression of ER was more prevalent in the Discovery group (*p*-value < 0.0001).

**Table 1:** Clinical characteristics of METABRIC Discovery and Validation data.

Characteristic	METABRIC Discovery	METABRIC Validation	P^†^
Age at diagnosis	61 (51, 70)^∗^	63 (52,71)	0.2107
CNA burden (%)	6.62 (3.00, 11.28)	7.39 (3.18, 13.39)	0.0119
Subtype			0.0005
Normal	58 (6%)^‡^	144 (15%)	
LumA	454 (46%)	255 (26%)	
LumB	266 (27%)	222 (23%)	
Her2	84 (9%)	153 (15%)	
Basal	118 (12%)	211 (21%)	
Grade			0.0095
1	68 (7%)	98 (11%)	
2	407 (42%)	356 (40%)	
3	505 (51%)	444 (49%)	
ER-expr	784 (80%)	712 (72%)	<0.0001
PR-expr	517 (53%)	517 (52%)	0.9282
Her2-expr	112 (11%)	132 (13%)	0.1940

^∗^*For continuous variables (Age, CNA burden), quantiles [50th percentile (25th percentile, 75th percentile)] were presented. ^†^*p*-values were determined by Wilcoxon rank sum test for continuous variables and Fisher’s exact test for categorical variables. ^‡^The number of patients in each category and its proportion were presented. In ER-expr, PR-expr and Her2-expr, only the number of positive cases were presented*.

**FIGURE 1:**
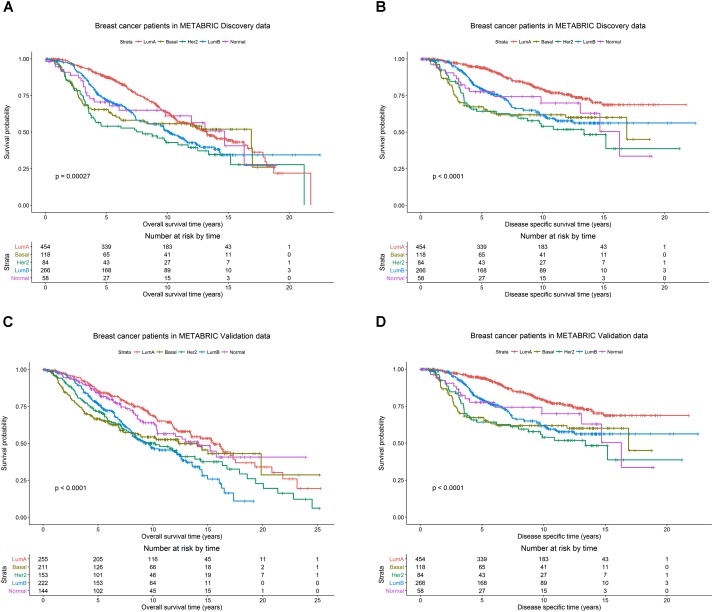
Breast cancer survival outcomes according to PAM50 subtypes. The *p*-value for log-rank test and a table counting the number of patients at risk are shown for each below case. Overall survival (OS) and disease specific survival (DSS) were used as surrogates for outcome in the METABRIC Discovery and Validation data sets, respectively. Kaplan-Meier plots for **(A)** OS for PAM50 subtypes in METABRIC Discovery data. **(B)** DSS for PAM50 subtypes in METABRIC Discovery data. **(C)** OS for PAM50 subtypes in METABRIC Validation data. **(D)** DSS for PAM50 subtypes in METABRIC Validation data.

The patients in the Discovery and Validation groups showed similar overall survival (OS) and disease specific survival (DSS) (**Figure 1**). OS and DSS were also examined among the PAM50 subtypes in the Discovery (**Figures 1A,B**) and Validation (**Figures 1C,D**) groups, respectively. As expected, the luminal A subtype had better OS and DSS rates compared to other subtypes, while basal-like and her2-enriched had the worst outcomes in both Discovery and Validation groups.

### CNA Landscape and Its Association With Breast Cancer Outcomes

**FIGURE 2:**
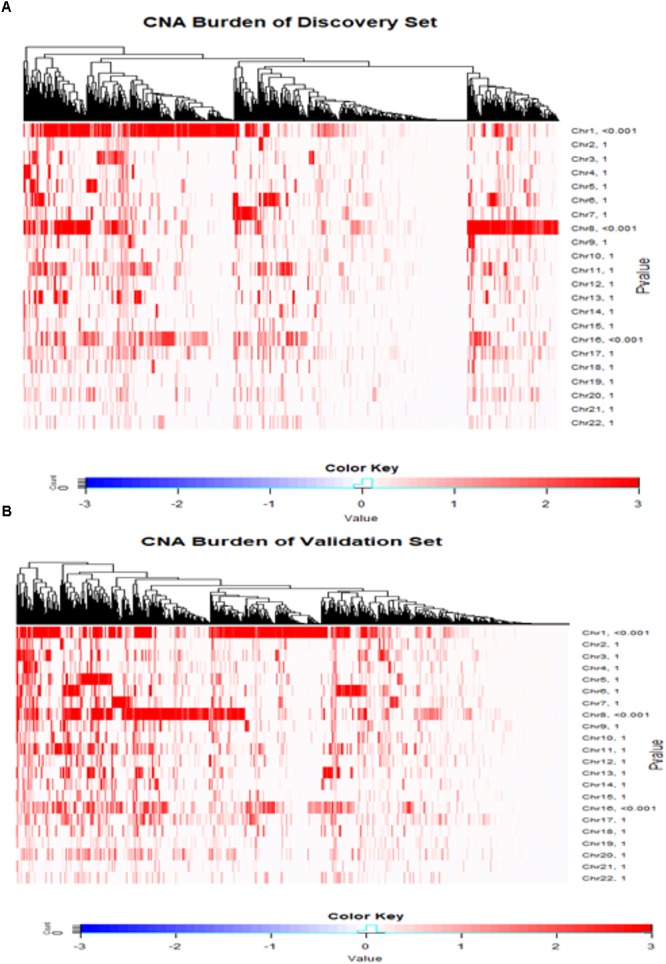
Copy number landscape of breast cancer. Heat maps of copy number alteration burden (%) in METABRIC Discovery **(A)** and Validation **(B)** data. The significance (*P*-value) of the CNA burden in each chromosome evaluated using one-sample Wilcoxon signed rank test was shown on the right side of the figures **(A,B)**. Chromosomes 1, 8 and 16 have significantly higher CAN burden than other chromosomes.

The CNA burdens of breast cancer genomes in METABRIC were visualized by heat map (**Figure 2**). CNA burdens on chromosomes 1, 8, and 16 were significantly higher than other chromosomes (*p*-value < 0.001) in both Discovery (**Figure 2A**) and Validation (**Figure 2B**) groups. For the top 10% most frequent genes on each chromosome separately for loss and gain groups, gene set enrichment analysis (**Supplementary Table S1**) showed that somatic aberrations on chromosome 1 interact closely with the genes involved in “regulation of humoral immune response (GO:002920).” Overexpression of immune responses against tumor associated antigens has been frequently reported in breast cancer patients (e.g., 82% antibodies against Her2/neu in cases with strong expression versus no antibodies in cases with weak expression) (Reuschenbach et al., 2009). Some somatic CNA-affected genes from chromosomes 1 belong to “MAPKAPK3_knockdown” related gene sets, which comprise genes involved in MAPK signaling pathways which provoke responses to mitogens and environmental stress stimuli (Lapin et al., 2014). Loss of genes such as MAPKAPK3 was reported in invasive breast carcinomas (Wei et al., 2015). One of the interesting gene sets affected by somatic CNAs on chromosome 8 is ‘MYC-MAX complex’ which comprises oncogenes deregulated in 50% of human cancers including breast cancer (Chen et al., 2018). Genes affected by somatic CNAs on chromosome 8 include members of ‘TP53 Receptors and Ligands’ gene set, which induce pro-apoptotic signaling in response to external stimuli via extrinsic apoptosis pathway (Wilson et al., 2013). In breast cancer, p53 mutation is strongly associated with disease severity and overall survival (Gasco et al., 2002). At last but not least, chromosome 16 also appeared to share important genes on somatic CNA segments with gene sets related to cell-cell junction interactions as well as kinase co-expression pathways. The genes for cadherin are frequently epigenetically deregulated in metastatic breast cancers (Andrews et al., 2012).

**FIGURE 3:**
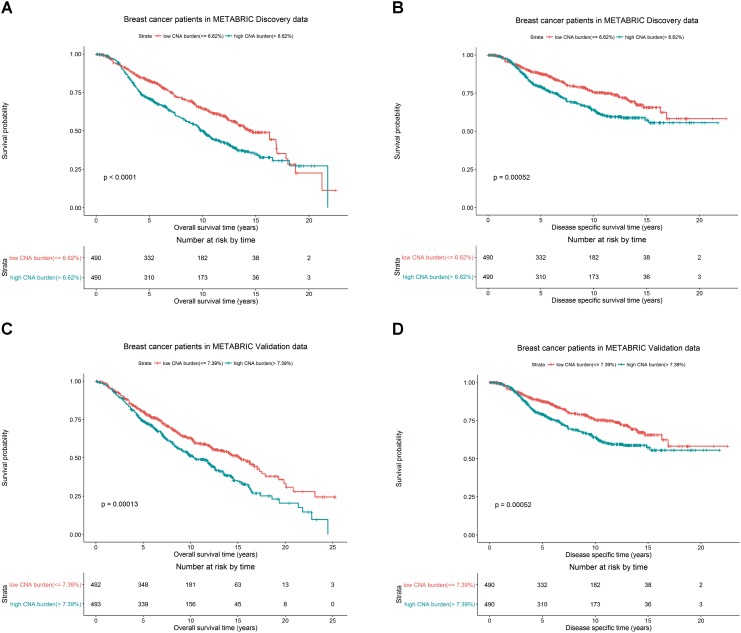
Breast cancer survival outcomes according to high and low CNA burden in METABRIC. The *p*-value for log-rank test and a table counting the number of patients at risk in each time point are shown in each case. High and low CNA burden is associated with OS and DSS of breast cancer in METABRIC. Kaplan–Meier plots for **(A)** OS for breast cancer by high and low CNA burden (cutoff 6.62%) in METABRIC Discovery data. **(B)** DSS for breast cancer by high and low CNA burden (cutoff 6.62%) in METABRIC Discovery data. **(C)** OS for breast cancer by high and low CNA burden (cutoff 7.39%) in METABRIC Validation data. **(D)** DSS for breast cancer by high and low CNA burden (cutoff 7.39%) in METABRIC Validation data.

We hypothesized whether CNA burden is associated with OS and DSS in METABRIC. The Kaplan-Meier estimators by high and low CNA burden in the Discovery and Validation groups were used as a preliminary test before we fit the data into more complicated models. It was revealed that high and low CNA burden indeed had a significant association with OS and DSS in Discovery (**Figures 3A,B**, both *p*-values < 0.001) and Validation (**Figures 3C,D**, *p*-values < 0.001 and = 0.003) groups. The significant association of the high and low CNA burden with OS was validated in TCGA data (**Supplementary Figure S1**, *p*-value < 0.05).

Additionally, in the Discovery group, patients with low CNA burden (<6.62%) had 82.7% 5-year OS rate and 87.7% 5-year DSS rate, better than the records for patients with high CNA burden(≥6.62%), who had 71.1% OS rate and 79.3% DSS rate. Similarly, in the Validation group, the patients with low CNA burden (<7.39%) had 80.2% 5-year OS rate and 85.1% 5-year DSS rate, while patients with high CNA burden (≥ 7.39%) had 74.1% OS rate and 81.6% DSS rate.

### CNA Burden by PAM50 Subtypes and Age

In the previous section, we reported a significant association between PAM50 subtypes and breast cancer survival in METABRIC patients. It is noteworthy that the strong association between the patient’s age and breast cancer survival has previously been reported in literature (Adami et al., 1986; Jenkins et al., 2014; Azim et al., 2015). Accordingly, in order to show that the reported association between breast cancer outcome and CNA burden did not result from the confounding effects of PAM50 subtypes or age, we investigated the association between CNA burden and PAM50 subtypes as well as between CNA burden and age.

**FIGURE 4:**
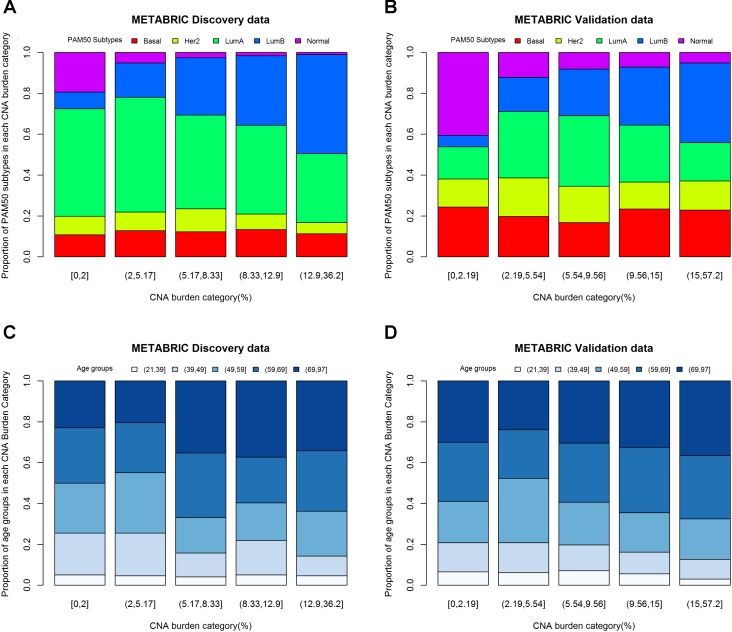
CNA burden by quintiles within PAM50 subtypes and age groups. **(A)** CNA burden by PAM50 subtypes in METABRIC Discovery data. **(B)** CNA burden by PAM50 subtypes in METABRIC Validation data. **(C)** CNA burden by age groups in METABRIC Discovery data. **(D)** CNA burden by age groups in METABRIC Validation data.

One-way ANOVA was conducted to compare the effect of PAM50 subtypes on CNA burden. The results of *F*-test from Discovery (*p*-value < 0.0001) and Validation (*p*-value < 0.0001) groups indicated that PAM50 subtypes had a significant association with CNA burden. Furthermore, the histograms of PAM50 subtypes across CNA burden categories (divided by its quintiles) showed that for both Discovery (**Figure 4A**) and Validation (**Figure 4B**) groups, the incidence of Luminal B tumors increased with CNA burden, whereas the incidence of Normal-like decreased (Chi-squared test *p*-value < 0.0001). Therefore, PAM50 subtypes were associated with CNA burden in METABRIC patients. Similar association result was also observed in TCGA patients (*p*-value < 0.001).

Considering the fact that both age and CNA burden are continues variables, we fitted a linear regression model for evaluating the effect of age on CNA burden. The results appeared to be significant for both Discovery (*p*-value for *F*-test = 0.0012) and Validation (*p*-value for *F*-test = 0.0020) groups. The estimates of the parameters for age were positive, which implies that older patients may have greater CNA burden. One-way ANOVA was also conducted to study the effect of age groups on CNA burden in 21–39, 40–49, 50–59, 60–69, and 70–97 years of age (Jenkins et al., 2014). The results were consistent with the linear regression model for both Discovery (*p*-value = 0.0052) and Validation (*p*-value = 0.0599) groups. Moreover, the histograms of age groups across CNA burden categories (**Figures 4C,D**) supported this association. The linear regression model of age on CNA burden in TCGA cohort also showed that they had a significant association (*p*-value for *F*-test = 0.04).

### The CNA Burden Is a Prognostic Factor in Breast Cancer Survival

In order to quantify the effects of CNA burden on OS and DSS in METABRIC patients, Cox proportional hazard regression models were fitted to the data. First we fitted the univariate Cox regression models between the CNA burden and OS, as well as CNA burden and DSS. Consequently, we found a significant association in both Discovery and Validation groups (*p*-value < 0.001) (**Table 2**). Furthermore, we estimated that hazard ratio in all cases was greater than one, which suggested that a higher CNA burden would result in a greater risk of death. These results were consistent with the observed outcomes from KM analysis by high and low CNA burden. Subsequently, in order to adjust the confounding effects of age and PAM50 subtypes, the two covariates were respectively and jointly included in multiple Cox regression models. The results suggested that age and PAM50 subtypes affect OS significantly, no matter if they were examined alone or jointly (*p*-value < 0.01) (**Table 2**). However, in case of DSS, the effect of age appeared to be insignificant (*p*-value > 0.05), while PAM50 was still significantly effective.

**Table 2:** Multiple time to event analysis for the association of CNA burden with OS and DSS, controlling for covariates.

	Overall survival^∗^								Disease specific survival^§^							
	METABRIC Discovery				METABRIC Validation				METABRIC Discovery				METABRIC Validation				
Variable in model	*P*^†^	HR^•^	95%	CI°	*P*	HR	95%	CI	*P*	HR	95%	CI	*P*	HR	95%	CI
CNA burden	<0.001	1.02	1.01	1.04	<0.001	1.03	1.01	1.04	<0.001	1.03	1.01	1.05	<0.001	1.03	1.01	1.04
CNA burden	<0.001	1.02	1.01	1.03	<0.001	1.02	1.01	1.03	<0.001	1.03	1.01	1.05	<0.001	1.03	1.01	1.04
Age at diagnosis	<0.001	1.02	1.02	1.03	<0.001	1.03	1.02	1.04	0.438	1	0.99	1.01	0.25	0.99	0.98	1.00
CNA burden	0.001	1.02	1.01	1.04	<0.001	1.02	1.01	1.03	0.004	1.03	1.01	1.04	0.008	1.02	1.01	1.03
PAM50 Subtypes																
Normal	0.12	1.43	0.91	2.24	0.257	1.22	0.87	1.71	0.002	2.34	1.36	4.03	0.012	1.99	1.16	3.41
LumA	Ref.^♢^	–	–		Ref.	–	–		Ref.	–	–		Ref.	–	–	
LumB	0.035	1.29	1.02	1.63	<0.001	1.61	1.23	2.11	<0.001	1.81	1.32	2.48	<0.001	2.83	1.82	4.42
Her2	<0.001	1.97	1.45	2.69	<0.001	1.68	1.26	2.23	<0.001	3.02	2.04	4.48	<0.001	4.15	2.67	6.47
Basal	0.088	1.31	0.96	1.79	0.003	1.53	1.15	2.02	<0.001	2.4	1.65	3.47	<0.001	3.56	2.30	5.51
CNA burden	0.005	1.02	1.01	1.03	<0.001	1.02	1.01	1.03	0.005	1.03	1.01	1.04	0.008	1.02	1.01	1.03
Age at diagnosis	<0.001	1.03	1.02	1.04	<0.001	1.04	1.03	1.04	0.756	1	0.99	1.01	0.925	0.99	0.99	1.01
PAM50 Subtypes																
Normal	0.026	1.67	1.06	2.63	0.013	2.18	1.09	2.18	0.002	2.36	1.37	4.07	0.014	1.98	1.15	3.41
LumA	Ref.^‡^	-	-		Ref.	-	-		Ref.	-	-		Ref.	-	-	
LumB	0.084	1.23	0.97	1.56	0.001	1.56	1.19	2.04	<0.001	1.8	1.31	2.47	<0.001	2.83	1.82	4.43
Her2	<0.001	2.19	1.61	3.00	<0.001	1.81	1.36	2.42	<0.001	3.04	2.05	4.51	<0.001	4.15	2.66	6.47
Basal	<0.001	1.67	1.25	2.34	<0.001	2.13	1.60	2.85	<0.001	2.43	1.65	3.57	<0.001	3.54	2.26	5.56

^∗^*METABRIC Discovery total *n* = 980, overall survival events *n* = 432, METABRIC Validation total *n* = 985, overall survival events *n* = 455. ^§^METABRIC Discovery disease specific events *n* = 260, METABRIC Validation disease specific events *n* = 244. ^†^*P*-value for the parameter’s significance. All *p*-values of multivariate cox models are smaller than or equal to 0.006. ^∙^Hazard ratio. °95% confidence interval. ^♢^Reference category. ^‡^The baseline category of PAM50 subtype in multivariable cox model*.

Eventually, the proportional hazard ratios were calculated based on the fitted models. The results implied that unlike CNA burden, both age and PAM50 subtypes were time dependent variables required to be eliminated from the Cox models (*p*-value < 0.05, which rejects the null hypothesis that the hazard ratio for these variables remain constant over time) (**Table 3**). Accordingly, we proceeded to stratified Cox models (**Table 4**). Once the covariates age and PAM50 subtypes were stratified and fitted in the models (either separately or jointly), their effects on OS and DSS were significant for both Discovery and Validation groups (**Table 4**). These results suggested that CNA burden has significant association with OS and DSS in METABRIC breast cancer patients.

**Table 3:** Assessment of proportional hazard assumptions for Cox models.

	Overall survival						Disease specific survival					
	METABRIC Discovery			METABRIC Validation			METABRIC Discovery			METABRIC Validation		
Variable in model	rho^†^	chisq^•^	*P*^§^	rho	chisq	*P*	rho	chisq	*P*	rho^†^	chisq	*P*
CNA burden	-0.06	1.43	0.23	0.04	0.73	0.39	<-0.01	<0.01	0.99	0.06	0.74	0.39
CNA burden	-0.09	3.14	0.08	0.03	0.29	0.59	-0.02	0.13	0.72	0.05	0.5	0.48
Age at diagnosis	0.21	28.75	<0.01	0.18	19.82	<0.01	0.21	15.37	<0.01	0.08	2.07	0.15
*Global*^∗^	-	30.44	<0.01	-	20.55	<0.01	-	15.37	<0.01	–	31.7	<0.01
CNA burden	-0.05	0.77	0.38	0.04	0.73	0.39	0.02	0.05	0.82	0.04	0.42	0.52
PAM50 Subtypes												
Normal	-0.15	9.59	<0.01	-0.03	0.45	0.5	-0.12	3.48	0.06	0.12	3.28	0.07
LumA	Ref.^♢^	–	–	Ref.	–	–	Ref.	–	–	Ref.	–	–
LumB	-0.13	7.38	0.01	<-0.01	0.03	0.87	-0.11	3.27	0.07	0.13	4.36	0.04
Her2	-0.23	22.54	<0.01	-0.11	5.8	0.02	-0.21	11.93	<0.01	-0.02	0.11	0.74
Basal	-0.32	43.53	<0.01	-0.25	28.23	<0.01	-0.4	41.01	0.15	-0.12	3.28	0.07
*Global*	–	59.39	<0.01	–	39.75	<0.01	–	45.88	<0.01	–	31.7	<0.01
CNA burden	-0.07	1.63	0.2	0.04	0.8	0.37	0.01	0.02	0.9	0.04	0.42	0.52
Age at diagnosis	0.15	13.51	<0.01	0.12	8.27	<0.01	0.11	3.97	0.04	-0.004	0.006	0.94
PAM50 Subtypes												
Normal	-0.13	6.7	<0.01	0.003	0.004	0.95	-0.11	2.76	0.1	0.12	3.18	0.07
LumA	Ref.	–	–	Ref.	–	–	Ref.	–	–	Ref.	–	–
LumB	-0.13	7.46	<0.01	-0.02	0.22	0.64	-0.12	3.77	0.05	0.13	4.38	0.04
Her2	-0.21	19.04	<0.01	-0.13	6.9	<0.01	-0.2	10.55	<0.01	-0.02	0.11	0.73
Basal	-0.29	34.1	<0.01	-0.23	20.66	<0.01	-0.36	32.18	<0.01	-0.12	3.17	0.07
*Global*	–	75.15	<0.01	–	50.45	<0.01	–	49.98	<0.01	–	31.7	<0.01

^†^*Pearson Correlation Coefficients between survival time and Scholenfeld Residuals. ^•^Chi-square statistics results. ^§^*p*-value of the null hypothesis that the proportional hazard assumption is valid for a variable. ^∗^Assessment results when taking all variables of a model into consideration. ^♢^Reference category. The baseline category of PAM50 subtype in multivariable cox model*.

**Table 4:** Multiple time to event analysis for the association of CNA burden with OS and DSS using stratified Cox models.

	Overall survival							Disease specific survival						
	METABRIC Discovery													
Variable in model	*P*	HR	95%	CI	rho	chisq	*P*	*P*	HR	95%	CI	rho	chisq	*P*
CNA burden	<0.001	1.02	1.01	1.04	-0.09	2.93	0.09	<0.001	1.03	1.01	1.05	-0.01	0.04	0.84
*Age at diagnosis (stratified)*	–	–	–		–	–	–	–	–	–		–	–	–
CNA burden	0.004	1.02	1.01	1.04	-0.08	2.21	0.14	0.007	1.03	1.01	1.04	0.01	0.02	0.88
PAM50 Subtypes (stratified)	–	–	–		–	–	–	–	–	–		–	–	–
CNA burden	0.009	1.02	1.01	1.04	-0.09	3.43	0.06	0.008	1.03	1.01	1.04	-0.007	0.01	0.92
Age at diagnosis(stratified)	–	–	–		–	–	–	–	–	–		–	–	–
PAM50 Subtypes (stratified)	–	–	–		–	–	–	–	–	–		–	–	–

				**METABRIC Validation**					

CNA burden	<0.001	1.02	1.01	1.03	0.02	0.2	0.66	<0.001	1.03	1.01	1.04	0.05	0.49	0.48
Age at diagnosis (stratified)	–	–	–		–	–	–	–	–	–		–	–	–
CNA burden	<0.001	1.02	1.01	1.03	0.04	0.85	0.36	0.011	1.02	1.00	1.03	0.44	0.45	0.5
PAM50 Subtypes (stratified)	–	–	–		–	–	–	–	–	–		–	–	–
CNA burden	<0.001	1.02	1.01	1.03	0.04	0.76	0.38	0.013	1.02	1.00	1.03	0.04	0.42	0.52
Age at diagnosis(stratified)	–	–	–		–	–	–	–	–	–		–	–	–
PAM50 Subtypes(stratified)	–	–	–		–	–	–	–	–	–		–	–	–

### The Analysis of CNA Burden and Breast Cancer Survival by Tumor Subtypes

The strong association between PAM50 subtypes and CNA burden encouraged us to generalize Cox models for each of the PAM50 subtypes. However, most of the models failed to test any significant association. In the Discovery group, CNA burden was associated with OS and DSS only for Her2-enriched subtype considered either alone (*p*-values = 0.013 and 0.013) or along with age (*p*-value = 0.02 and 0.003) (**Table 5**). A marginal significance was also observed for Luminal A in all cases except for DSS in the Validation group (*p*-value = 0.128).

**Table 5:** Cox proportional hazard model results in each PAM50 subtype.

Overall survival		METABRIC Discovery						METABRIC Validation					
	Variable	PAM50 subtype	Model P	P	HR	95%	CI	PAM50 subtype	Model P	P	HR	95%	CI
	CNA burden	Normal	0.185	0.133	1.06	0.98	1.15	Normal	0.006	0.391	1.02	0.98	1.06
	Age	*n1 = 22*^∗^		0.821	1.01	0.96	1.05	*n1 = 51*		0.003	1.04	1.01	1.06
	CNA burden	*n2 = 58*	0.071	0.079	1.07	0.99	1.15	*n2 = 144*	0.319	0.32	1.02	0.98	1.07
	CNA burden	LumA	< 0.001	0.208	1.02	0.99	1.04	LumA	<0.001	0.017	1.03	1.01	1.06
	Age	*n1 = 178*		< 0.001	1.05	1.04	1.07	*n1 = 103*		<0.001	1.1	1.07	1.12
	CNA burden	*n2 = 454*	0.058	0.059	1.03	1.00	1.05	*n2 = 255*	< 0.001	<0.001	1.04	1.02	1.07
	CNA burden	LumB	0.083	0.695	1.01	0.98	1.03	LumB	< 0.001	0.164	1.02	0.99	1.04
	Age	*n1 = 128*		0.029	1.02	1.00	1.04	*n1 = 119*		<0.001	1.04	1.02	1.06
	CNA burden	*n2 = 266*	0.663	0.663	1.01	0.98	1.03	*n2 = 222*	0.281	0.281	1.01	0.99	1.04
	CNA burden	Her2	0.042	0.020	1.07	1.01	1.13	Her2	0.778	0.486	1.01	0.98	1.04
	Age	*n1 = 52*		0.786	1.00	0.98	1.03	*n1 = 86*		0.879	1	0.98	1.02
	CNA burden	*n2 = 84*	0.012	0.013	1.07	1.01	1.13	*n2 = 153*	0.489	0.489	1.01	0.98	1.04
	CNA burden	Basal	0.343	0.209	1.02	0.99	1.05	Basal	0.014	0.093	1.02	0.99	1.03
	Age	*n1 = 52*		0.394	1.01	0.99	1.03	*n1 = 96*		0.027	1.02	1.00	1.04
	CNA burden	*n2 = 118*	0.234	0.235	1.02	0.99	1.05	*n2 = 211*	0.058	0.059	1.02	0.99	1.04
Disease specific survival	CNA burden	Normal	0.856	0.753	1.02	0.90	1.16	Normal	0.515	0.255	1.04	0.98	1.10
	Age	*n1 = 16*		0.604	0.99	0.94	1.04	*n1 = 25*		0.993	1	0.97	1.03
	CNA burden	*n2 = 58*	0.840	0.840	1.01	0.90	1.14	*n2 = 144*	0.249	0.252	1.04	0.98	1.10
	CNA burden	LumA	0.002	0.063	1.04	1.00	1.07	LumA	0.009	0.245	1.03	0.98	1.08
	Age	*n1 = 81*		0.005	1.03	1.01	1.05	*n1 = 29*		0.008	1.05	1.01	1.09
	CNA burden	*n2 = 454*	0.031	0.031	1.04	1.00	1.08	*n2 = 255*	0.127	0.128	1.04	0.99	1.09
	CNA burden	LumB	0.802	0.623	1.01	0.98	1.04	LumB	0.751	0.776	1.01	0.97	1.04
	Age	*n1 = 84*		0.663	1.00	0.99	1.02	*n1 = 64*		0.47	1.01	0.98	1.04
	CNA burden	*n2 = 266*	0.614	0.614	1.01	0.98	1.04	*n2 = 222*	0.823	0.823	1	0.97	1.04
	CNA burden	Her2	0.003	0.003	1.11	1.04	1.18	Her2	0.076	0.227	1.02	0.99	1.05
	Age	*n1 = 36*		0.020	0.97	0.94	0.99	*n1 = 60*		0.044	0.98	0.96	0.99
	CNA burden	*n2 = 84*	0.012	0.013	1.08	1.02	1.15	*n2 = 153*	0.281	0.282	1.02	0.99	1.05
	CNA burden	Basal	0.320	0.310	1.02	0.99	1.05	Basal	0.118	0.048	1.02	1.00	1.04
	Age	*n1 = 43*		0.297	0.99	0.96	1.01	*n1 = 66*		0.439	0.99	0.97	1.01
	CNA burden	*n2 = 118*	0.274	0.276	1.02	0.99	1.05	*n2 = 211*	0.054	0.055	1.02	0.99	1.04

^∗^*n1 stands for the number of events in each PAM50 subtypes, n2 stands for the number of total patients in each PAM50 subtypes*.

## Conclusion and Discussions

In this study, we examined the CNA burden of patients from METABRIC study and highlighted chromosomes 1, 8, and 16 to carry the highest burden. We showed an association between PAM50 subtypes and CNA burden, as the incidence of Luminal B tumors increases with CNA burden while the incidence of Normal-like tumors decreases. We also reported a relationship between age and CNA burden since older people tend to have higher CNA burden. Furthermore, we proposed CNA burden as a prognostic criterion for estimating OS and DSS of breast cancer in METABRIC patients, as our analysis showed that CNA burden has a significant association with both OS and DSS in our stratified Cox models.

While studying the overall survival of METABRIC patients, we stratified the patients by PAM50 subtypes and 45-year age bound, and observed a significant association between changes in CNA burden and hazard ratio. Per each 1% change in CNA burden the same changes in different subtypes would occur, however each group is still distinctive as it has its own baseline hazard function. Regarding disease specific survival, we found no confounding effect for age. Accordingly, each PAM50 subtype will have its own baseline function while the effect of CNA burden on hazard ratio remains the same. We also showed that all measured CNA hazard ratios to be greater than one, which evidently showed that higher CNA burden would result in worse outcomes in METABRIC patients. Additionally, the prognostic effect of CNA burden is not salient in all PAM50 subtypes. We observed that only HER2-enriched and Luminal A tumors followed such a trend. Therefore, CNA burden and PAM50 subtypes have a joint prognostic effect on OS and DSS in METABRIC patients.

We defined CNA burden as a percentage of the CNA segments found in a cancer genome and based all our examinations on it. This definition is empowered by considering the size of CNA segments, monitoring the whole amount of genome and studying the fraction of genome which is encountered with CNAs. However, it is prone to some limitations resulting from the cutoffs used to define a region as a gain or loss such as the region’s size and the number of probes found in a region. Other definitions proposed in literature suggest “the number of CNA segments” as the criterion for defining a CNA burden. However, this explanation is also limited by incapability of considering the size of CNA segments in an individual.

As a final remark, the association between CNA burden and breast cancer survival is a novel concept with limited investigations. Consequently, it calls for more studies through future works in order to reveal the relationship between CNA burden and other tumor characteristics and its effect on breast cancer survival.

## Ethics Statement

The study did not generate any human-specific data. It used the data set published before, which is publicly available online.

## Author Contributions

PH supervised the study. PH and LZ designed the experiments. LZ performed the statistical analysis. LZ and NF drafted the manuscript. CC helped in preparing the data. All authors critically revised the manuscript.

## Conflict of Interest Statement

The authors declare that the research was conducted in the absence of any commercial or financial relationships that could be construed as a potential conflict of interest.

## Supplementary Material

FIGURE S1 Breast cancer survival outcomes according to high and low CNA burden in TCGA. The *p*-value for log-rank test and a table counting the number of patients at risk in each time point are shown in each case. High and low CNA burden is associated with OS of breast cancer in TCGA.

TABLE S1 Gene set enrichment analysis results. We filtered the top 10% most frequent genes on each of chromosomes 1, 8 and 16 separately for loss and gain groups.
