# Supplementary figures and images for: Association Analysis of Somatic Copy Number Alteration Burden With Breast Cancer Survival
Source: Front Genet. 2018 Oct 1;9:421. doi: 10.3389/fgene.2018.00421 (PMC6178888; doi:10.3389/fgene.2018.00421)

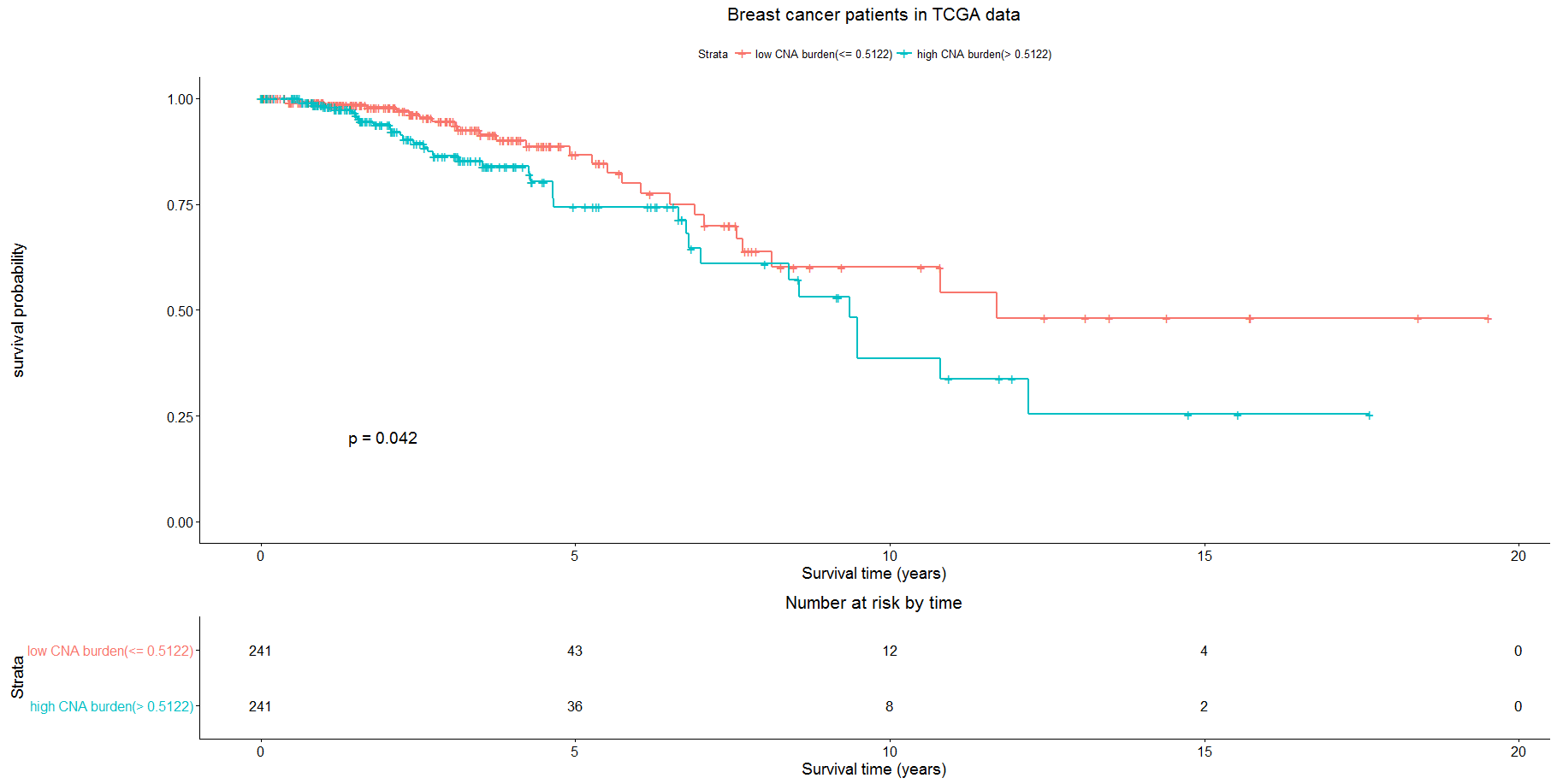

Supplement: FIGURE S1 — Breast cancer survival outcomes according to high and low CNA burden in TCGA. The p-value for log-rank test and a table counting the number of patients at risk in each time point are shown in each case. High and low CNA burden is associated with OS of breast cancer in TCGA. [file Image_1.TIFF]
